# Parameter Estimation for the Intravoxel Incoherent Motion-diffusional Kurtosis Imaging Model Using Synthetic Q-space Learning Toward Breast Tumor Diagnosis

**DOI:** 10.2463/mrms.mp.2026-0045

**Published:** 2026-08-19

**Authors:** Kousei Konya, Yuki Ichinoseki, Erina Kato, Naoko Mori, Hideki Ota, Shunji Mugikura, Kei Takase, Yoshitaka Masutani

**Affiliations:** 1 Department of Medical Image Computation, Tohoku University Graduate School of Medicine, Sendai, Miyagi, Japan; 2 Department of Diagnostic Radiology, Tohoku University Hospital, Sendai, Miyagi, Japan; 3 Department of Radiology, Akita University Graduate School of Medicine, Akita, Akita, Japan; 4 Division of Image Statistics, Tohoku Medical Megabank Organization, Tohoku University, Sendai, Miyagi, Japan; 5 Department of Diagnostic Radiology, Tohoku University Graduate School of Medicine, Sendai, Miyagi, Japan

**Keywords:** breast magnetic resonance imaging, diffusion magnetic resonance imaging, intravoxel incoherent motion–diffusional kurtosis imaging, machine learning, parameter estimation

## Abstract

**Purpose:**

Synthetic Q-space learning (synQSL), which uses only synthetic data in the training of regressors, has demonstrated promising results in parameter estimation for brain diffusion MRI (dMRI). This study aimed to evaluate the performance of synQSL in breast dMRI for intravoxel incoherent motion–diffusional kurtosis imaging (IVIM–DKI) parameter estimation, with comparing several types of regressors and conventional fitting by nonlinear least squares fitting (NL-LSF).

**Methods:**

In synthesizing data for synQSL, IVIM–DKI parameters were sampled from uniform distributions and substituted into the signal model along with b-values to generate diffusion-weighted imaging (DWI) signals. In addition, Rician noise was mixed to the signals. We prepared datasets of $10^{5}$ and $10^{6}$ samples, and trained multi-layer perceptron (MLP), Kolmogorov–Arnold networks (KAN), and random forest (RF) regressors for synQSL. The performance of the parameter estimation methods including NL-LSF was evaluated using a digital phantom including various value combinations of IVIM–DKI parameters and clinical data of 67 cases (13 benign and 54 malignant lesions) through quantitative analysis and visual assessment.

**Results:**

In the digital phantom, the regressors of synQSL achieved significantly lower root mean square error (RMSE) for f, $D^{*}$, and D than NL-LSF (P<0.05) with less variation among the parameter sets. In clinical datasets, synQSL not only improved the visual quality of parameter maps but also showed significant differences between benign and malignant lesions in parameters while the NL-LSF failed. Furthermore, the estimation times for all synQSL regressors were substantially shorter than that of NL-LSF. Among the synQSL regressors, MLP showed superior properties including computational cost.

**Conclusion:**

synQSL demonstrated superior parameter estimation performance compared to NL-LSF in breast IVIM–DKI analysis. In this study, MLP was considered the most suitable regressor for synQSL among those we examined, based on the balance between estimation accuracy and computational costs.

## Introduction

Breast cancer is the most diagnosed cancer in women.^1^ Currently, mammography is widely used for breast cancer screening and diagnosis. However, its sensitivity decreases significantly in patients with dense breasts.^2^ To address this limitation, the role of breast MRI has been expanding. Dynamic contrast-enhanced MRI has been reported to have high sensitivities of 94%–100%, whereas its specificity varies widely around 37%–97%.^3^ Furthermore, the use of contrast agents may be contraindicated or require careful administration in patients with renal failure, heart disease, or pregnancy.^4^ From these perspectives, diffusion-weighted imaging (DWI) has gained increasing importance in the characterization of breast lesions. Diffusion MRI (dMRI) is a modality to quantitatively characterize tissue microstructure using DWIs acquired with different b-values, which reflect strengths of motion-probing gradient. The apparent diffusion coefficient (ADC), calculated by fitting the signal intensities of DWI with different b-values to a mono-exponential model, has been reported to be useful for distinguishing between benign and malignant breast lesions.^5^^,^^6^ However, the mono-exponential model has limitations in separating the perfusion component and reflecting restricted diffusion due to tissue microstructure.^7^ These limitations led to the proposal of advanced signal models such as intravoxel incoherent motion (IVIM),^7^ which separates diffusion and perfusion effects, and diffusional kurtosis imaging (DKI),^8^ which captures non-Gaussian diffusion. The IVIM–DKI model integrates these 2 models to enable simultaneous assessment of both effects.^9^ Several studies have reported the utility of parameters derived from these models for differentiating benign and malignant breast tumors.^10^^–^^14^

For estimating IVIM–DKI parameters, nonlinear least squares fitting (NL-LSF) is commonly used, but 2 issues have been pointed out.^15^ The first is the high computational cost due to iterative improvement of parameters. The second is the high sensitivity to noise and dependency on initial parameter values, which leads to unstable estimation.^15^^–^^17^ To improve the estimation quality of IVIM–DKI parameters, 2 main strategies are generally considered: the application of denoising techniques and the use of advanced estimation methods. The examples for the former, several approaches were proposed and have shown their merits to some extent.^18^^,^^19^ Regarding the latter, segmented fitting^15^^,^^20^^,^^21^ and Bayesian approaches^22^ have been proposed. Although these methods have improved the estimation performance, they have not fully resolved the challenges.

Recently, for the further advanced methods, machine learning–based approaches have emerged in brain dMRI. For example, Golkov et al. proposed Q-space deep learning,^23^ which learns the mapping from DWI signals to model parameters. However, a significant problem in machine learning approaches using real images for training is the difficulty in obtaining diverse data with ground truth. To address this, a synthetic approach, which uses only synthetic data to train the regressor, has been proposed.^24^^,^^25^ This approach, known as synthetic Q-space learning (synQSL), has demonstrated promising results compared to NL-LSF.^26^ So far, applications of machine learning in the field of breast dMRI have primarily focused on tumor detection^27^ and classification^28^^,^^29^, and its application to parameter estimation remains limited. Applying synQSL to parameter estimation for breast tissue is expected to resolve the challenges of NL-LSF.

Therefore, the purpose of this study was to evaluate IVIM–DKI parameter estimation performance in breast dMRI by synQSL.

## Materials and Methods

In this section, we describe the data synthesis for training and tests, the digital phantom and clinical MRI datasets, the regressor construction for synQSL, and the evaluation and statistical analysis.

### Data synthesis for training and tests

The DWI signal value $S\left(b\right)$ of the IVIM–DKI model is expressed by the following formula:

(1) $$S\left(b\right)≅S_{0}\left\{\;f\text{exp}\left(-bD^{*}\right)+\left(1-f\right)\text{exp}\left(-bD+\frac{1}{6}b^{2}D^{2}K\right)\right\},$$

where $S_{0}$ is the baseline signal value, f is the volume fraction of the perfusion component, b is the b-value, $D^{*}$ is the diffusion coefficient of the perfusion component, D is the diffusion coefficient, and K is the diffusional kurtosis.

Data synthesis for training and test in synQSL was performed in the following steps. IVIM–DKI parameters were sampled from uniform distributions within the following physiologically plausible ranges: $S_{0}$, 500–8000; f, 0.0–1.0; $D^{*}$, 0.5–3.5 $\left(\times 10^{-2}mm^{2}/s\right)$; D, 0.2–3.0 $\left(\times 10^{-3}mm^{2}/s\right)$; and K, 0.0–4.0. Additionally, the constraint of D×K≤ 3/$b_{max}$ was applied to ensure monotonic signal decay up to the maximum b-value ($b_{max}=$ 2500 $s/mm^{2}$).^8^ These parameters were substituted into Eq. (1) together with the b-values. Rician noise^16^^,^^17^ was then added to the signal values using a simplified version of the noise model as the following formula^8^^,^^25^:

(2) $$S^{\prime }=\sqrt{S^{2}+N{\left(0,\sigma \right)}^{2}},$$

where $S^{\prime }$ is the noise-corrupted value, S is the original signal value from Eq. (1), $N\left(0,\sigma \right)$ is the zero-mean Gaussian random value with a standard deviation (SD) of σ. The noise level was controlled by the noise ratio (NR), defined as $NR=\sigma /S_{0}^{mdn}$, where $S_{0}^{mdn}$ is the median value of $S_{0}$. Through this process, synthetic datasets ($\;\;f,\;D^{*},D,K,S_{n}^{{\;}^{\prime }}\;\left[n=0,\ldots ,\;6\right]$) for training, validation, and test were generated at three noise levels (NR = 0.001, 0.010, 0.100).

### Digital phantom

The spatial distribution of IVIM–DKI parameters is illustrated in Fig. 1. The entire volume (128 × 128 × 128 voxels) was partitioned into 64 blocks (32 × 32 × 32 voxels) by dividing each side (x, y, z) into 4 equal parts. Different combinations of f, $D^{*}$, and D were assigned to these blocks. Within each block, 27 sub-blocks (8 × 8 × 8 voxels) with different K values were arranged. The spatially distributed parameters were substituted into Eq. (1) along with $S_{0}=4250$ and b-values, thereby generating the digital phantom.

### Patients and MRI acquisition

This retrospective study was approved by the Ethics Committee of Tohoku University Hospital (registration #: 2023-1-804). The study population consisted of 67 cases, including 13 benign and 54 malignant lesions. An experienced radiologist manually delineated the lesion regions on DWIs. The lesion diameters were 4.92–56.25 (mean ± SD: 20.99 ± 15.69) mm and 5.63–35.86 (mean ± SD: 15.87 ± 7.33) mm in benign and malignant lesions, respectively. MRI examinations were performed using a 3.0T MR scanner (Achieva dStream; Philips, Best, The Netherlands) equipped with a 16-channel breast coil. DWI was acquired using 2D echo-planar imaging sequence. The acquisition parameters were as follows: TR/TE, 5300/80 ms; FOV, 180 × 180 $mm^{2}$; matrix size, 256 × 256; number of excitations, 2; slice thickness, 5.0 mm; and b-values, 0, 50, 850, 1000, 1500, 2000, and 2500 $s/mm^{2}$.

### Regressor construction for synQSL

We used multi-layer perceptron (MLP),^30^ Kolmogorov–Arnold networks (KAN),^31^ and random forest (RF)^32^ as regressors for synQSL. We constructed an independent regressor for each IVIM–DKI parameter, configured to accept 6 logarithmic signal decay ratios ($ln\left(S_{n}^{{\;}^{\prime }}/S_{0}^{{\;}^{\prime }}\right)$, where n=1,…, 6) as input and predict a single parameter. The training dataset consisted of $10^{6}$ samples for MLP and RF, whereas $10^{5}$ samples were used for KAN due to memory constraints. Hyperparameters for each regressor were optimized using a grid search, and the configuration yielding the lowest root mean square error (RMSE) on the validation data was selected for the evaluation experiments. Table 1 shows the search ranges of hyperparameters.

### Evaluation and statistical analysis

We performed cross-tests on synthetic test data ($10^{6}$ samples) to evaluate RMSE for all combinations of training and test noise levels. Subsequently, parameter estimation by NL-LSF and synQSL trained at NR=0.100 was performed on the digital phantom with Rician noise added to obtain NR=0.100 (Fig. 2) and clinical datasets of breast dMRI. This noise level was selected based on preliminary experiments to reduce a potential noise-level mismatch between the training data and clinical data. We compared the parameter maps estimated by applying regressors trained with the 3 *NR*s ​​mentioned above to clinical data. As a result, NR=0.100, which showed fewer physiologically implausible values and better visualization of anatomical structures, was considered the most reasonable training noise level for the clinical data in this study.

In the digital phantom analysis, we examined the 24 regions of parameter sets (12 benign and 12 malignant) approximating the IVIM–DKI parameter values of breast tumors reported in previous studies,^33^^,^^34^ excluding other unrealistic parameter combinations. Each parameter set contained 512 pixels. The parameter sets for benign tumors consisted of f=0.11, combined with $D^{*}=1.5,and\;\;2.0\;\left(\times 10^{-2}mm^{2}/s\right)$, *D* = 1.3, $and\;\;1.8\;\left(\times 10^{-3}mm^{2}/s\right)$, and *K* = 0.22, 0.33, and 0.44. The parameter sets for malignant tumors consisted of $D=0.8\;\left(\times 10^{-3}mm^{2}/s\right)$, combined with f=0.11,and0.33, $D^{*}=1.5,and\;\;2.0\;\left(\times 10^{-2}mm^{2}/s\right)$, and *K* = 0.67, 0.78 and 0.89. The RMSE value was calculated for each parameter-set region, and the resulting 24 values were used for method comparisons. Additionally, estimated parameter distributions were compared within each region. The Wilcoxon signed-rank test with Bonferroni correction was used for both analyses. For the estimated-parameter distribution comparisons, effect sizes were calculated as $r=\left|Z\right|/\sqrt{N}$, where Z is the standardized test statistic from the Wilcoxon signed-rank test and N is the number of paired observations, because the large sample size could make even small differences statistically significant.

For the clinical data analysis, mean values of the IVIM–DKI parameters were calculated from ROIs placed along the lesion contours, and the quality of the parameter maps was visually evaluated. The mean values were compared between benign and malignant lesions using the Mann–Whitney U test. Comparisons among the estimation methods were also performed separately for benign and malignant lesions using the Wilcoxon signed-rank test with Bonferroni correction. Statistical significance was set at P<0.05 for all statistical tests. Furthermore, we measured the training time and estimation time to assess computational efficiency.

## Results

For all regressors, the lowest RMSE values in the cross-tests were observed when the noise ratios of the training and test data matched (Fig. 3), consistent with the previous study.^25^

In the digital phantom analysis, NL-LSF exhibited significantly higher RMSE than synQSL for f, $D^{*}$, and D (P<0.05) (Fig. 4a–4c). No significant differences were observed among the synQSL regressors. In contrast, there was no significant difference between NL-LSF and synQSL for K (Fig. 4d). However, KAN showed significantly higher RMSE compared to MLP and RF (P<0.05). Across all IVIM–DKI parameters, NL-LSF exhibited a large variation of RMSE values relative to synQSL. For the estimated parameter distributions, the synQSL regressors generally yielded estimated parameter values closer to the reference values than NL-LSF. As shown in the typical results in Fig. 5, NL-LSF tended to underestimate f and overestimate $D^{*}$, D, and K. However, in some parameter sets, NL-LSF showed similar or slightly better performance. Significant differences in the estimated parameter distributions were observed in many parameter-set regions, and the effect sizes tended to be larger for comparisons between NL-LSF and the synQSL regressors than for comparisons among the synQSL regressors (Supplementary Table 1).

In clinical data analysis, malignant lesions generally exhibited higher f, $D^{*}$, and K values, and lower D values compared to benign lesions (Fig. 6). These trends are consistent with previous studies,^33^^,^^34^ capturing the characteristics of malignancy associated with increased angiogenesis and high cellular density. Significant differences were confirmed for D and K across all methods (P<0.05) (Fig. 6c and 6d). Significant differences for f were observed with all synQSL regressors (Fig. 6a), whereas for $D^{*}$, a significant difference was found only with RF (P<0.05) (Fig. 6b). Method comparisons showed significant differences between NL-LSF and synQSL for most parameters, except for $D^{*}$ in benign lesions. Among the synQSL regressors, significant differences varied depending on the parameter and lesion group, with no clear consistent pattern (Supplementary Table 2). In the IVIM–DKI parameter maps, synQSL effectively suppressed the instability observed in the NL-LSF maps across the entire breast and demonstrated consistent visual characteristics among its regressors (Fig. 7). Based on the statistically significant difference for f observed only with synQSL (Fig. 6a), Fig. 8 displays magnified views of representative DWI and corresponding f maps in 3 breast lesions. NL-LSF yielded overall low values, resulting in maps with poor tissue contrast, whereas synQSL provided relatively clearer contrast.

In addition to estimation performance, computational costs were compared among the methods with the environments summarized in Supplementary Table 3. The computation times required to train a regressor under a specific hyperparameter configuration were approximately 5 minutes each for MLP and KAN, and 45 minutes for RF. It should be noted that $10^{5}$ samples were used for training KAN while $10^{6}$ for MLP and RF. When $10^{5}$ samples were used for MLP and RF, they were 1 minute and 5 minutes, respectively. Parameter estimation per clinical case was completed in approximately 80 minutes by NL-LSF, 2 minutes by MLP, 1 minute by KAN, and 2 minutes by RF.

## Discussion

In this study, we investigated the IVIM–DKI parameter estimation performance of synQSL in the breast region using synthetic test data, the digital phantom, and clinical data.

NL-LSF has been reported to be sensitive to noise in DWIs, leading to estimation instability.^15^^–^^17^ This instability was reflected in the digital phantom experiments, where NL-LSF showed higher RMSE values and larger RMSE variation than synQSL for most IVIM–DKI parameters (Fig. 4). The estimated parameter distribution–based analysis further supported this interpretation, as the values estimated by synQSL were generally closer to the reference values, whereas those obtained using NL-LSF tended to deviate from the reference values (Fig. 5). For the parameter ranges in which the superiority of synQSL against NL-LSF seems uncertain, we consider additional and adaptive training data for the improvement. The superiority of synQSL was also suggested by the clinical data. Significant differences in f between benign and malignant lesions were detected only with the synQSL regressors (Fig. 6a), and the corresponding parameter map showed clearer lesion contrast with synQSL than with NL-LSF (Fig. 8). These findings suggest that synQSL may provide more accurate and robust IVIM–DKI parameter estimation relative to NL-LSF. However, the interpretation of the $D^{*}$ results in the benign–malignant comparison requires caution (Fig. 6b). Although a significant difference was observed only with RF, the P-value for RF was only slightly below the significance level, and the other estimation methods did not show significant differences (P = 0.058, 0.16, 0.13, and 0.024 for NL-LSF, MLP, KAN and RF, respectively). Therefore, this significant difference should not be directly interpreted as evidence of superior differentiation ability of RF for $D^{*}$. Further validation with a larger and more balanced clinical dataset is needed to clarify the difference in $D^{*}$ between benign and malignant lesions. From a practical perspective, synQSL also demonstrated superior computational efficiency compared with NL-LSF. Therefore, synQSL may be a promising approach for IVIM–DKI parameter estimation in clinical breast lesion analysis.

In the comparison among regressors, KAN exhibited significantly higher RMSE for K than MLP and RF (Fig. 4d). This result might suggest that KAN in this study failed to adequately learn the complex non-linear relationship between the input signals and K values due to the constraint on training data size.

Not only for KAN, we are investigating 2 approaches to improve the estimation accuracy of synQSL under limited data conditions. The first approach is training data optimization based on mathematical analyses of submanifolds formed by dMRI models.^35^ These analyses are expected to provide adaptive training data synthesis, for reducing total quantity of the datasets. The second approach is the application of synthetic X-Q space learning (synXQSL),^36^ which incorporates spatial information from neighboring voxels into synQSL. synXQSL has been demonstrated to improve parameter estimation under high-noise conditions. These approaches are expected to further improve estimation performance even with limited amounts of training data.

MLP exhibited a shorter training time than RF and KAN when the training data samples were matched. These differences in training time could be attributed to the algorithmic characteristics of each regressor and the employed computational environments. In RF, training involves constructing multiple decision trees through repeated feature-based splits and is primarily executed on Central Processing Units (CPUs), which may result in longer training times. KAN employs learnable activation functions based on B-splines on each edge. During training, multiple spline coefficients must be updated simultaneously, increasing the computational burden, even with Graphics Processing Unit (GPU)–based parallelization. In contrast, MLP consists of linear layers with simple nonlinear activations. Gradient calculations for weights and biases are computationally straightforward and can be efficiently performed using parallelized matrix operations. Therefore, MLP had the lowest computational cost during training in this study.

Several studies have reported that high diagnostic performance in the differentiation of benign and malignant tumors is achieved by methods that use DWIs directly as input for convolutional neural networks.^28^^,^^29^ While these deep learning–based approaches offer workflow efficiency, they often suffer from the “black box” problem, where the specific image features or physical basis driving the diagnostic decision remain unclear. In contrast, radiomics approaches^37^ based on dMRI parameter maps can offer greater interpretability. Since the input features (e.g., histogram metrics) are directly linked to specific biophysical properties, it becomes possible to explicitly interpret the features contributing significantly to the diagnostic decision. However, the reliability of these features depends heavily on the quality of parameter maps. Therefore, the superior estimation performance of synQSL demonstrated in this study may serve as a critical foundation for realizing physiologically interpretable computer-aided diagnosis.

Our study has several limitations. First, as mentioned above, the training conditions for KAN were restricted by memory capacity. While we are investigating approaches such as training data optimization and synXQSL to improve estimation performance under limited data conditions, the imbalance in training data volume remains a fundamental limitation of this study. In general, parameter estimation performance may be influenced by several factors, including the size of the training data, the mismatch in noise levels between training and test data, and the characteristics of the target parameters. Although it is difficult to quantitatively disentangle the contribution of each factor, the difference in training data volume should be considered when interpreting the comparative performance of the regressors. Previous studies have reported that increasing the training data size improves estimation accuracy for MLP and RF,^38^ and a similar trend may be expected for KAN. Therefore, upgrading the computational environment to enable larger-scale training is expected to enhance KAN’s performance and allow a more rigorous comparison among the regressors in future studies. Second, synQSL entails a high computational cost for training and tuning. In this study, training a single regressor with 1 set of hyperparameters required approximately 4–45 minutes. Thus, the total time required for regressor tuning becomes substantial. Currently, breast DWI is performed using diverse magnetic field strengths and acquisition parameters across institutions. Applying synQSL necessitates optimizing regressors specifically tailored to each facility’s acquisition conditions, which imposes a significant burden. One solution for this problem is imaging standardization. For instance, the European Society of Breast Imaging has recently promoted the standardization of breast MRI protocols.^39^ As this standardization becomes widespread, it is expected that a common regressor could be applied across institutions, thereby dramatically improving the clinical applicability of synQSL. Finally, this study was limited to evaluating the IVIM–DKI parameter estimation performance of NL-LSF and synQSL. The impact of these estimation results on tumor differentiation requires further investigation. As future work, we intend to construct a classifier using the parameters estimated by synQSL and to quantitatively evaluate its diagnostic performance.

## Conclusion

In this study, we applied synQSL using MLP, KAN, and RF as regressors to IVIM–DKI parameter estimation in breast dMRI and evaluated their estimation performance in comparison with NL-LSF. Our results demonstrated that synQSL outperformed NL-LSF in terms of estimation accuracy, robustness, and computational efficiency. Therefore, synQSL is a promising tool for breast IVIM–DKI analysis. Currently, considering the balance between parameter estimation performance and computational costs, MLP could be the most suitable among the 3 regressors.

## Figures and Tables

**Supplementary Table 1:**
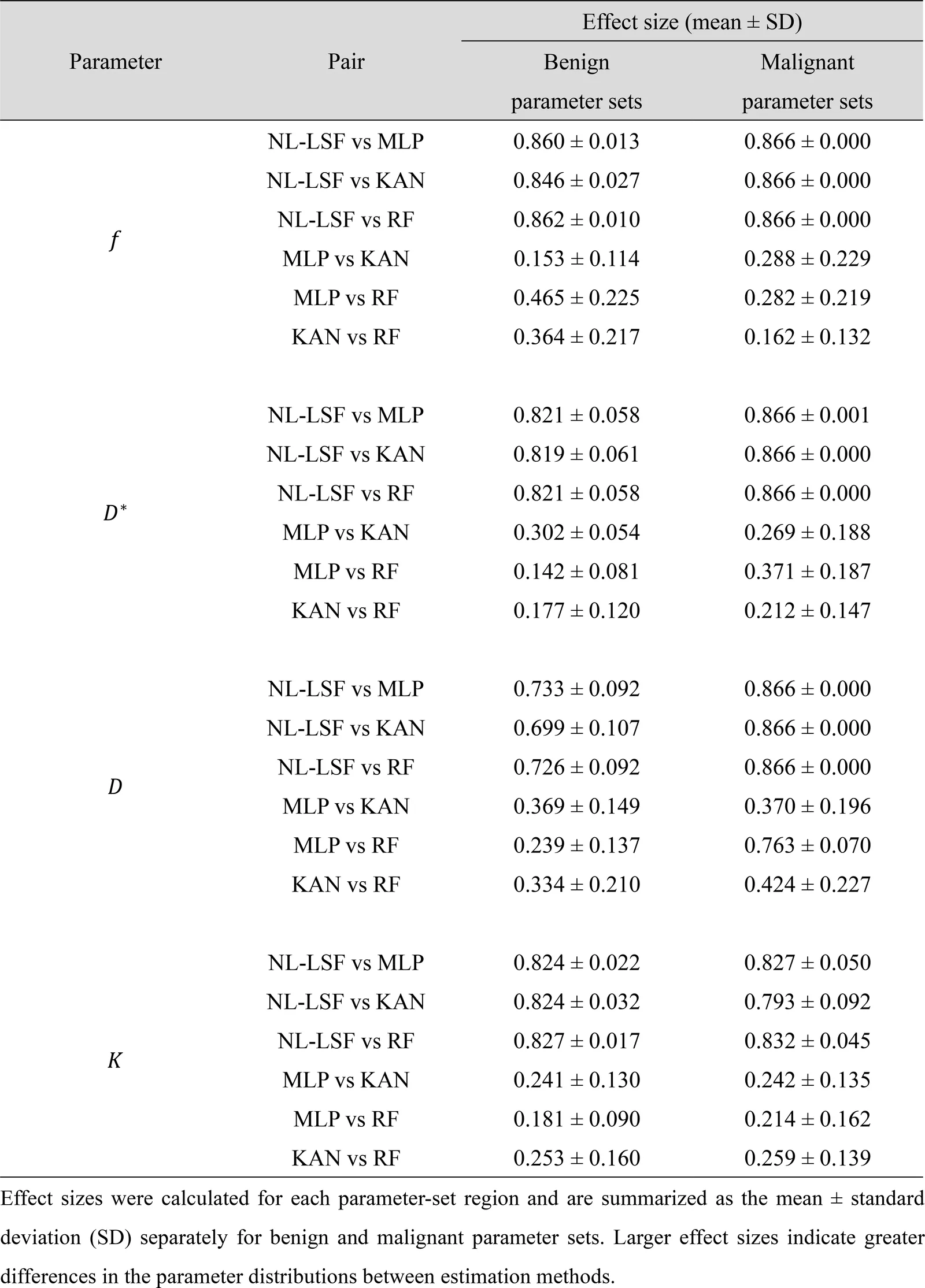
Effect sizes for method comparisons of IVIM-DKI parameter distributions in the digital phantom analysis

**Supplementary Table 2:**
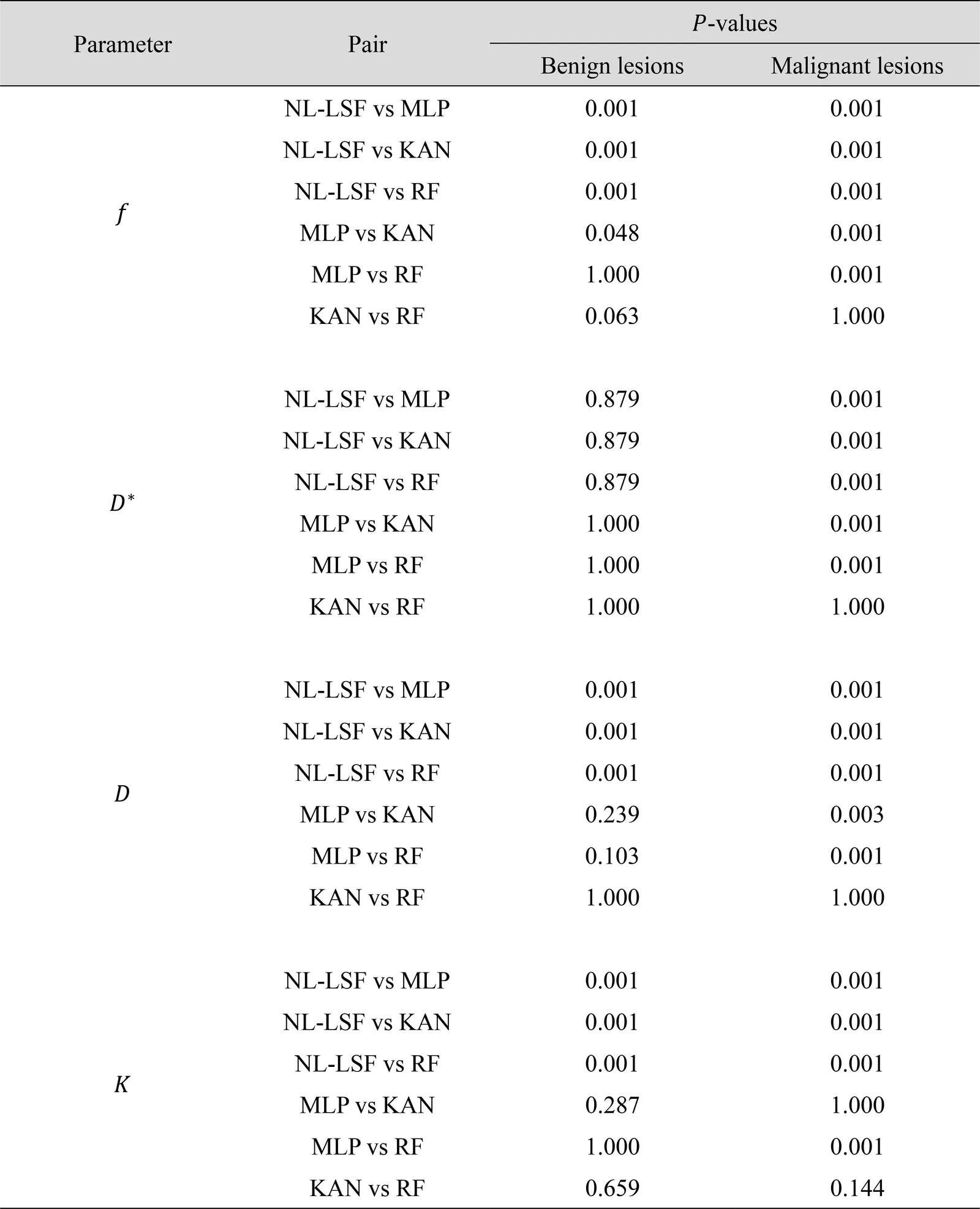
Bonferroni-corrected *P*-values for method comparisons in clinical data

**Supplementary Table 3:**
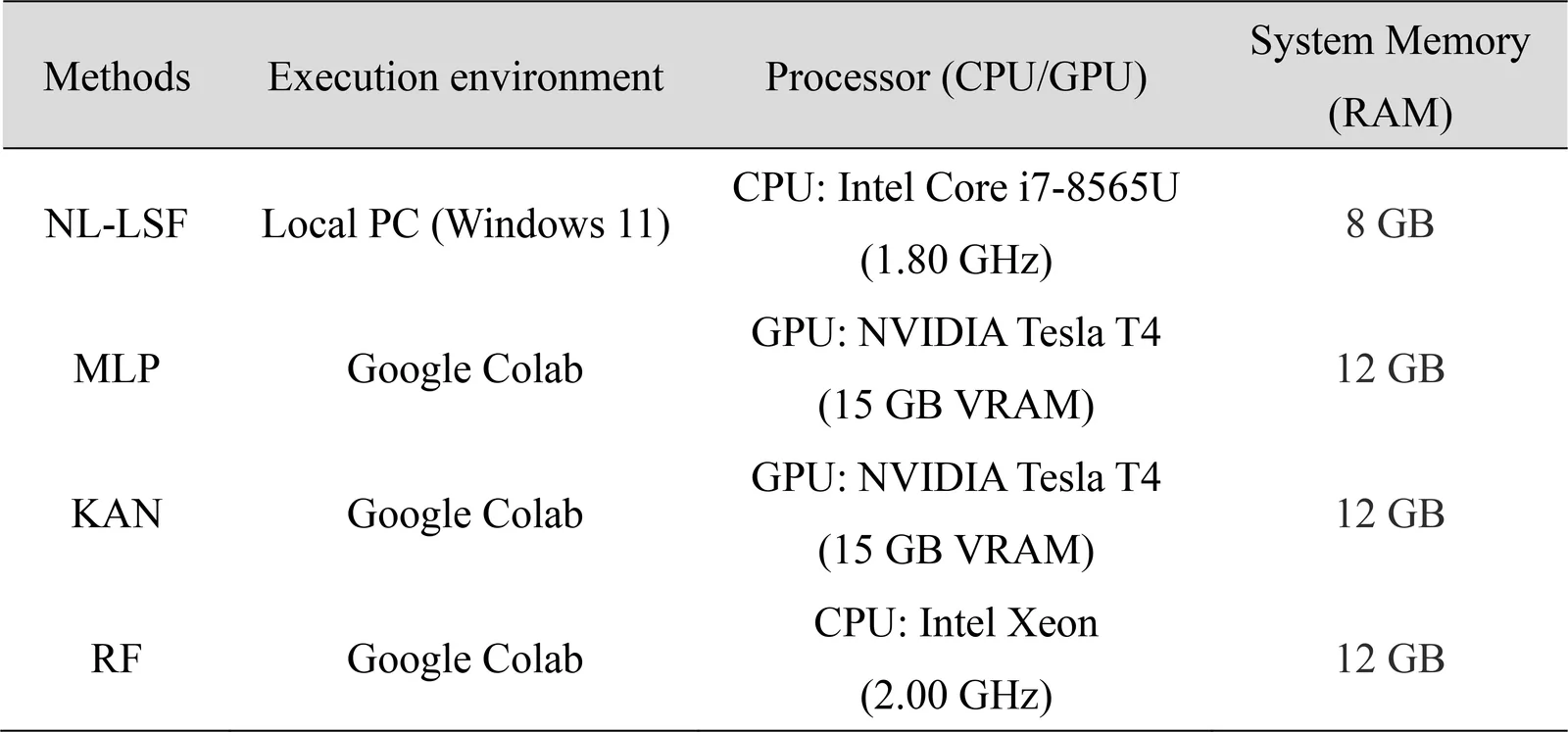
Computational environments for each estimation method

**Fig. 1:**
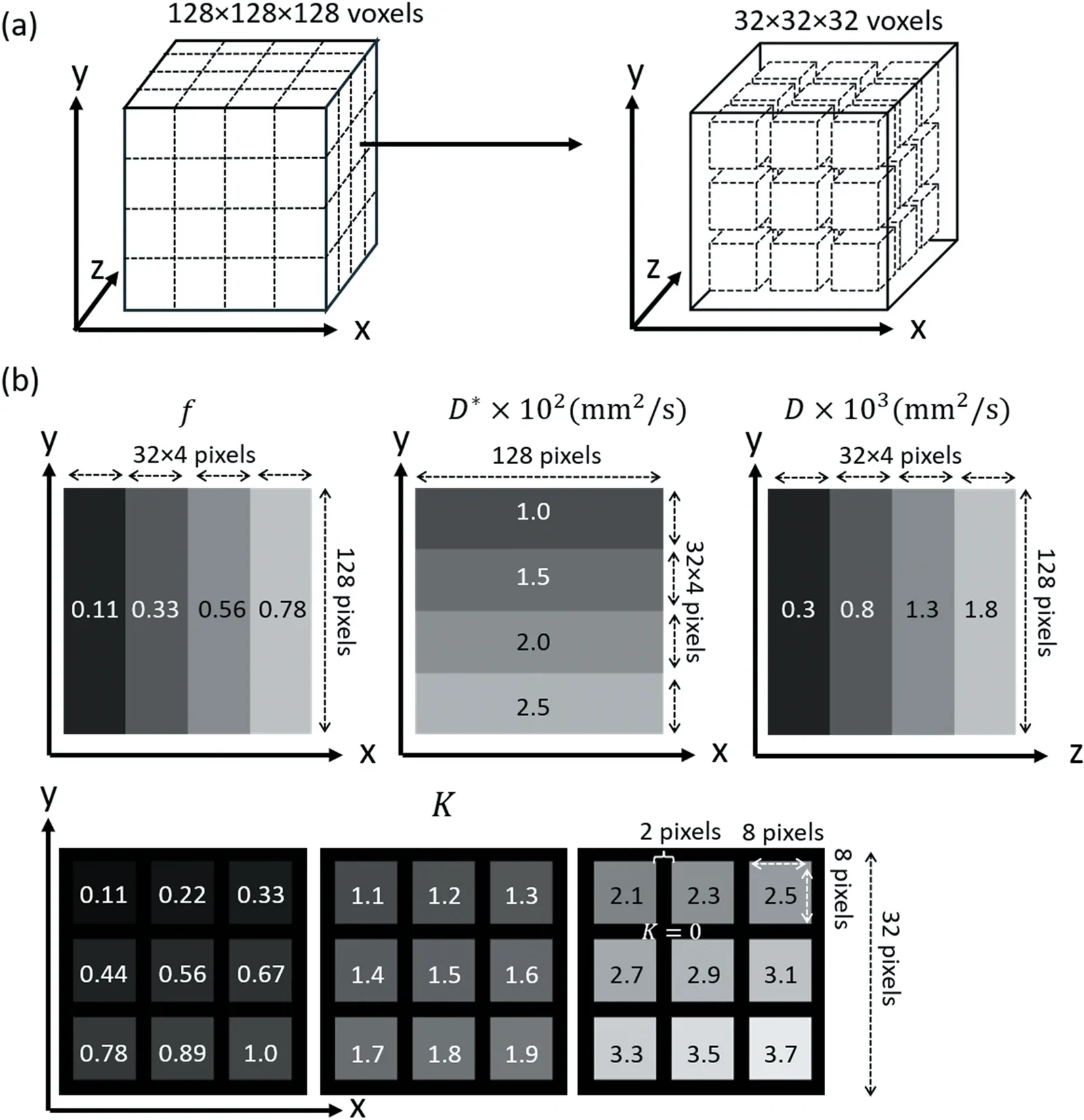
Spatial distribution of the IVIM–DKI parameters in the digital phantom. (**a**) Spatial configuration of the IVIM–DKI parameters, showing the division of the entire volume into 64 blocks and further subdivision into 27 sub-blocks within each block. (**b**) Assigned values of the IVIM–DKI parameters (f, $D^{*}$, D, and K). The margin area around the sub-blocks is set to K=0. IVIM–DKI, intravoxel incoherent motion–diffusional kurtosis imaging.

**Fig. 2:**
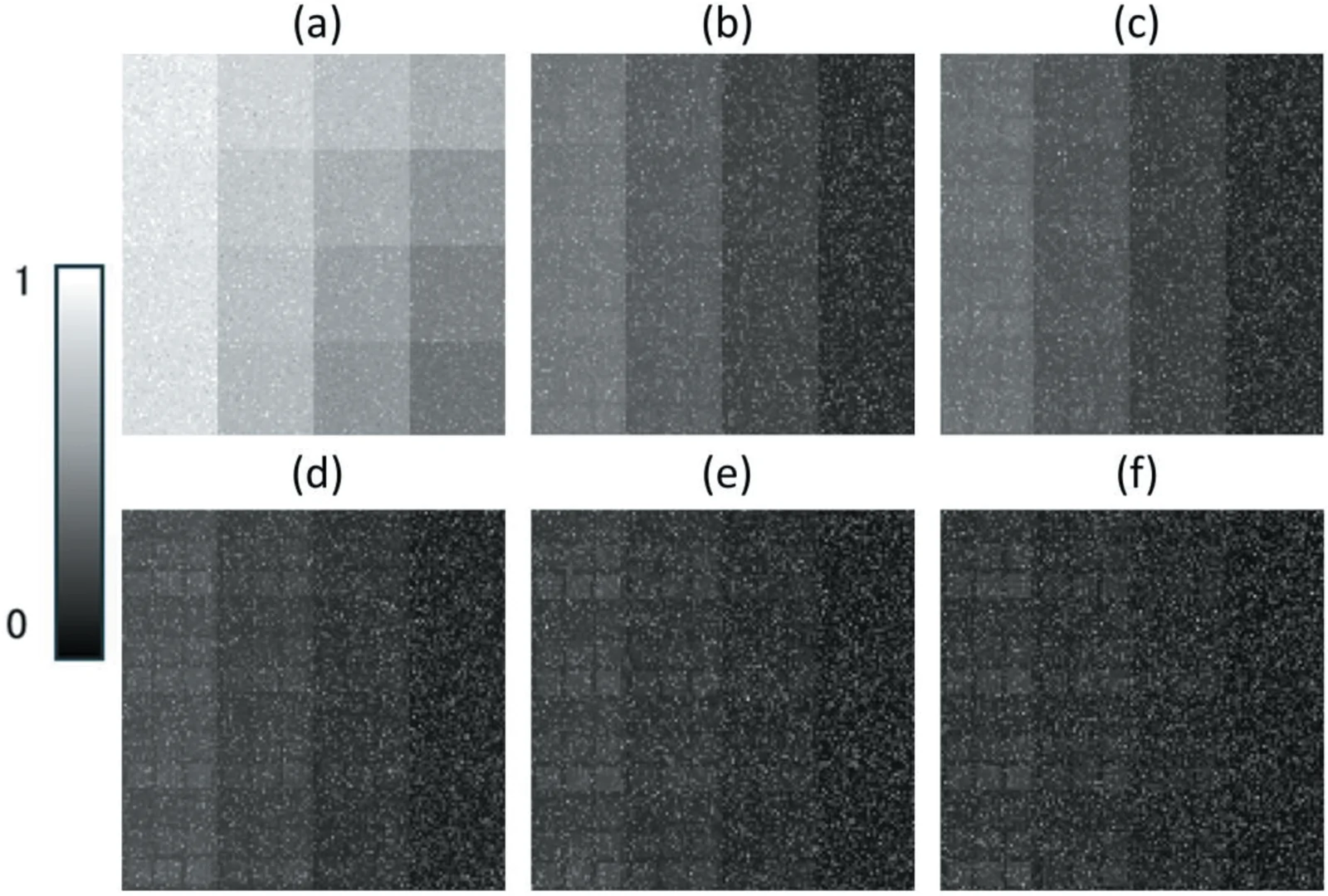
Digital phantom DWIs with NR=0.100. (**a**–**f**) Signal decay ratios ($S{\;}^{{\;}^{\prime }}\;\;/S_{0}\;\;\;\;{\text{\textbackslash{}1}}^{{\;}^{\prime }}$) at b-values of 50, 850, 1000, 1500, 2000, and 2500 $s/mm^{2}$, respectively. DWI, diffusion-weighted imaging; NR, noise ratio.

**Fig. 3:**
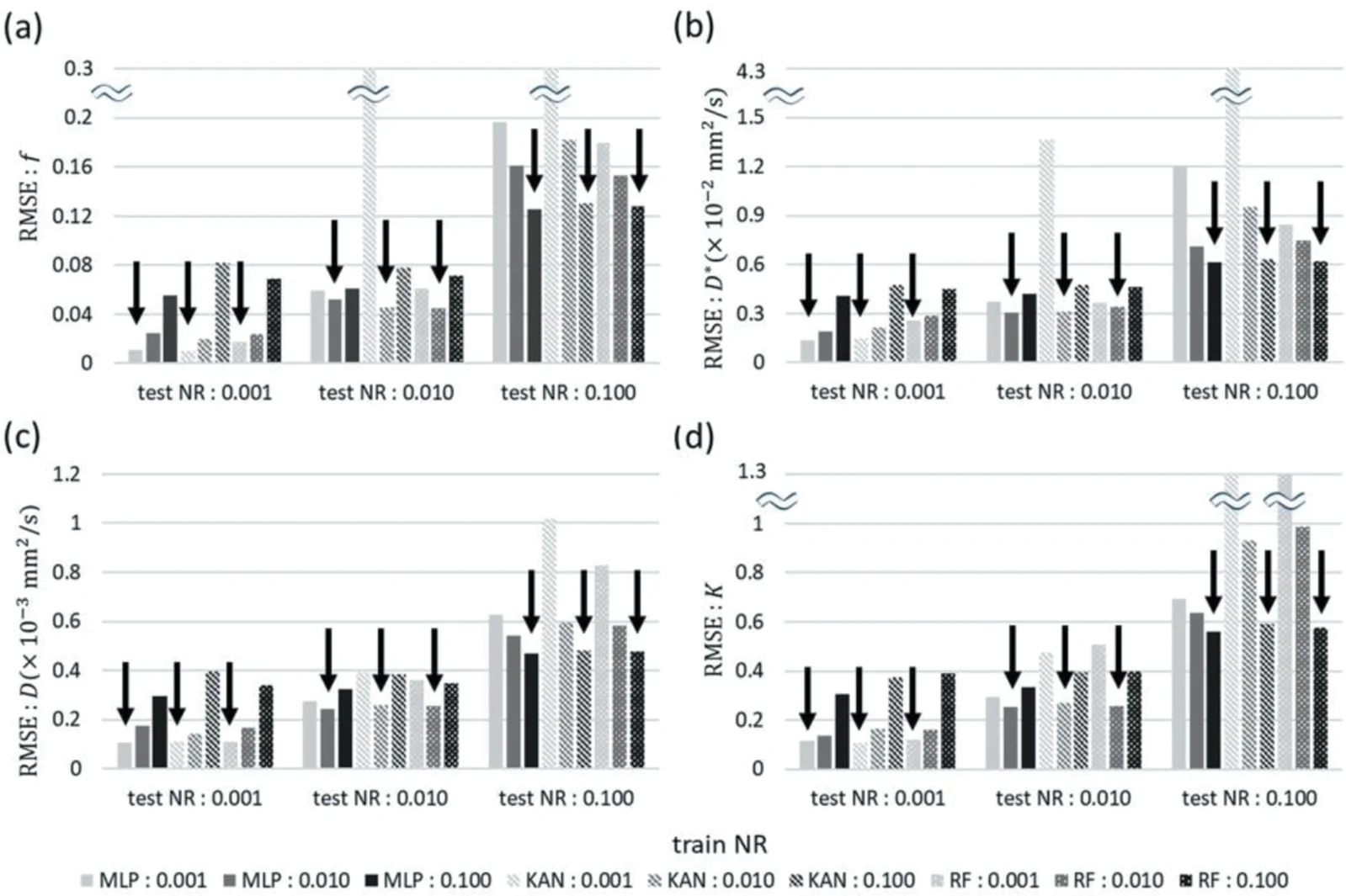
Cross-tests for parameter estimation on synthetic test data. RMSE of the synQSL regressors (MLP, KAN, and RF) for (**a**) f, (**b**)$\;D^{*}$, (**c**) D, and (**d**) K. KAN, Kolmogorov-Arnold networks; MLP, multi-layer perceptron; NR, noise ratio; RF, random forest: RMSE, root mean square error; synQSL, synthetic Q-space learning.

**Fig. 4:**
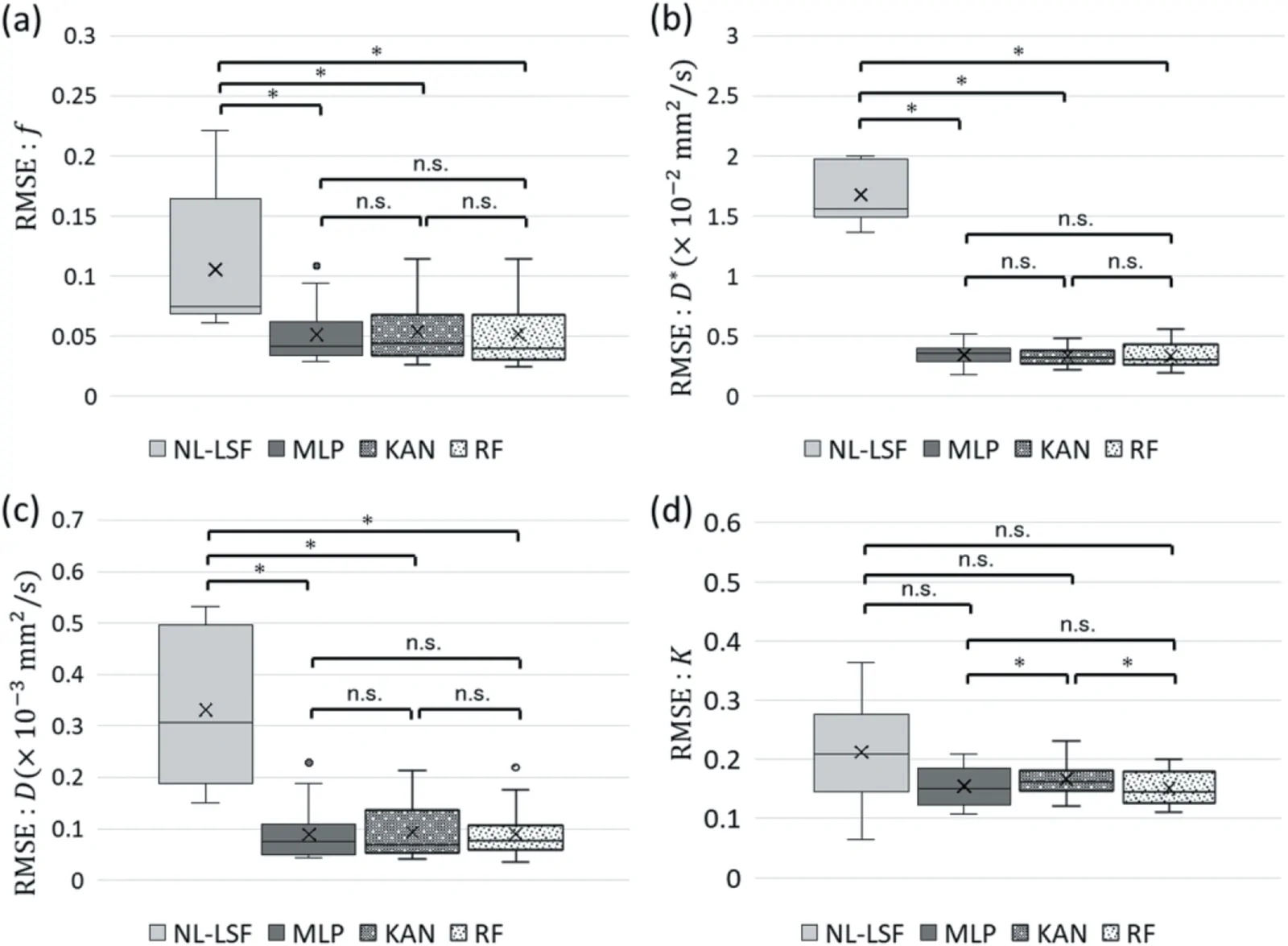
Boxplots of RMSE statistics in parameter estimation tests for the 24 parameter sets in the digital phantom. Comparison of RMSE for (**a**) f, (**b**)$\;D^{*}$, (**c**) D, and (**d**) K between NL-LSF and synQSL (MLP, KAN, and RF). *, p<0.05. KAN, Kolmogorov–Arnold networks; MLP, multi-layer perceptron; n.s., not significant; NL-LSF, nonlinear least squares fitting; RF, random forest; RMSE; synQSL, synthetic Q-space learning.

**Fig. 5:**
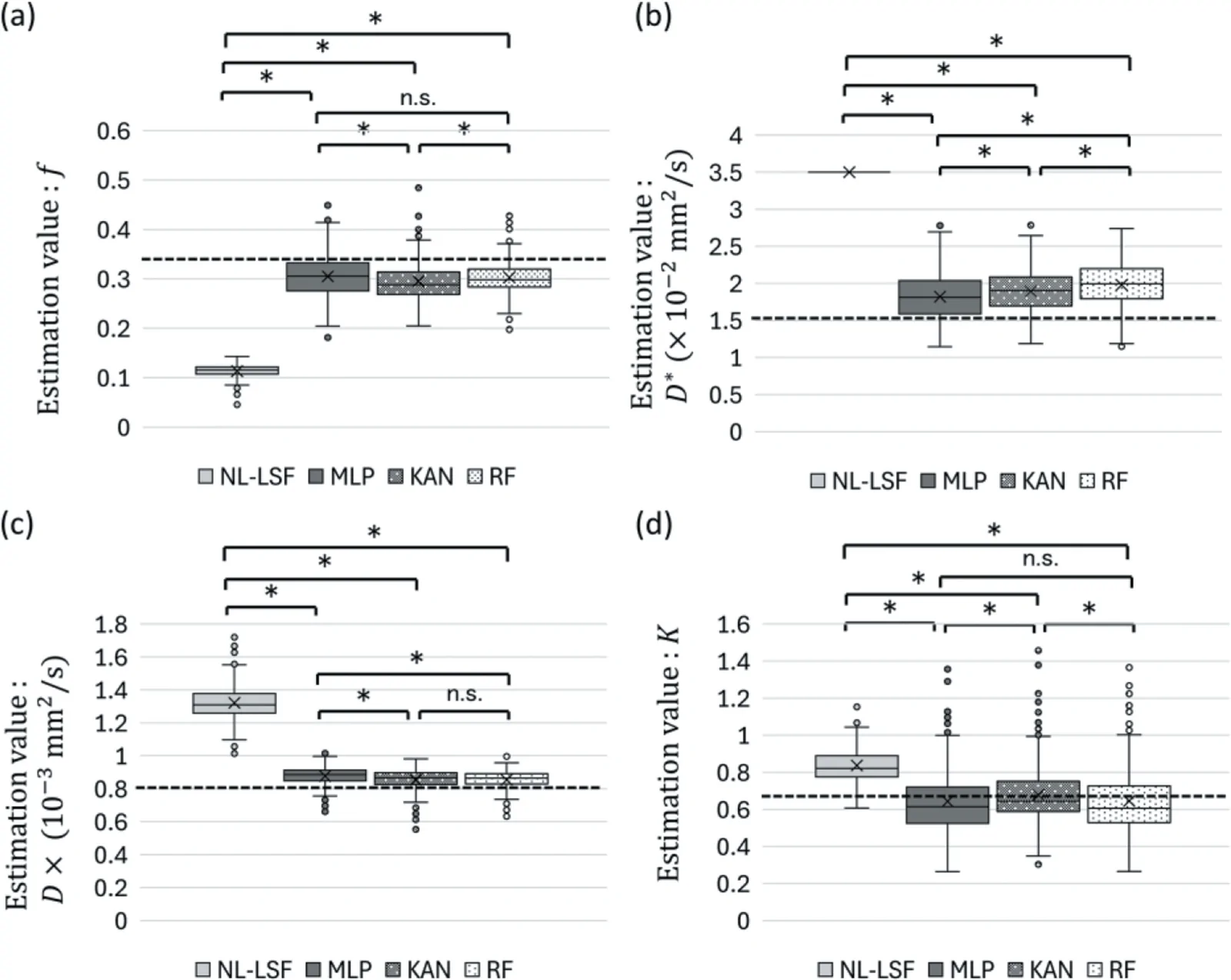
Boxplots of IVIM–DKI parameter values estimated by NL-LSF and synQSL for a representative parameter set. Boxplots are shown for (**a**) f, (**b**) $D^{*}$, (**c**) D, and (**d**) K. The dashed horizontal lines indicate the reference values: f=0.33, $D^{*}=1.5\;\left(\times 10^{-2}\;mm^{2}/s\right)$, $D=0.8\;\left(\times 10^{-3}\;mm^{2}/s\right)$, and K=0.667. *, P<0.05. IVIM–DKI, intravoxel incoherent motion–diffusional kurtosis imaging; KAN, Kolmogorov–Arnold networks; MLP, multi-layer perceptron; n.s., not significant; NL-LSF, nonlinear least squares fitting; RF, random forest; synQSL, synthetic Q-space learning.

**Fig. 6:**
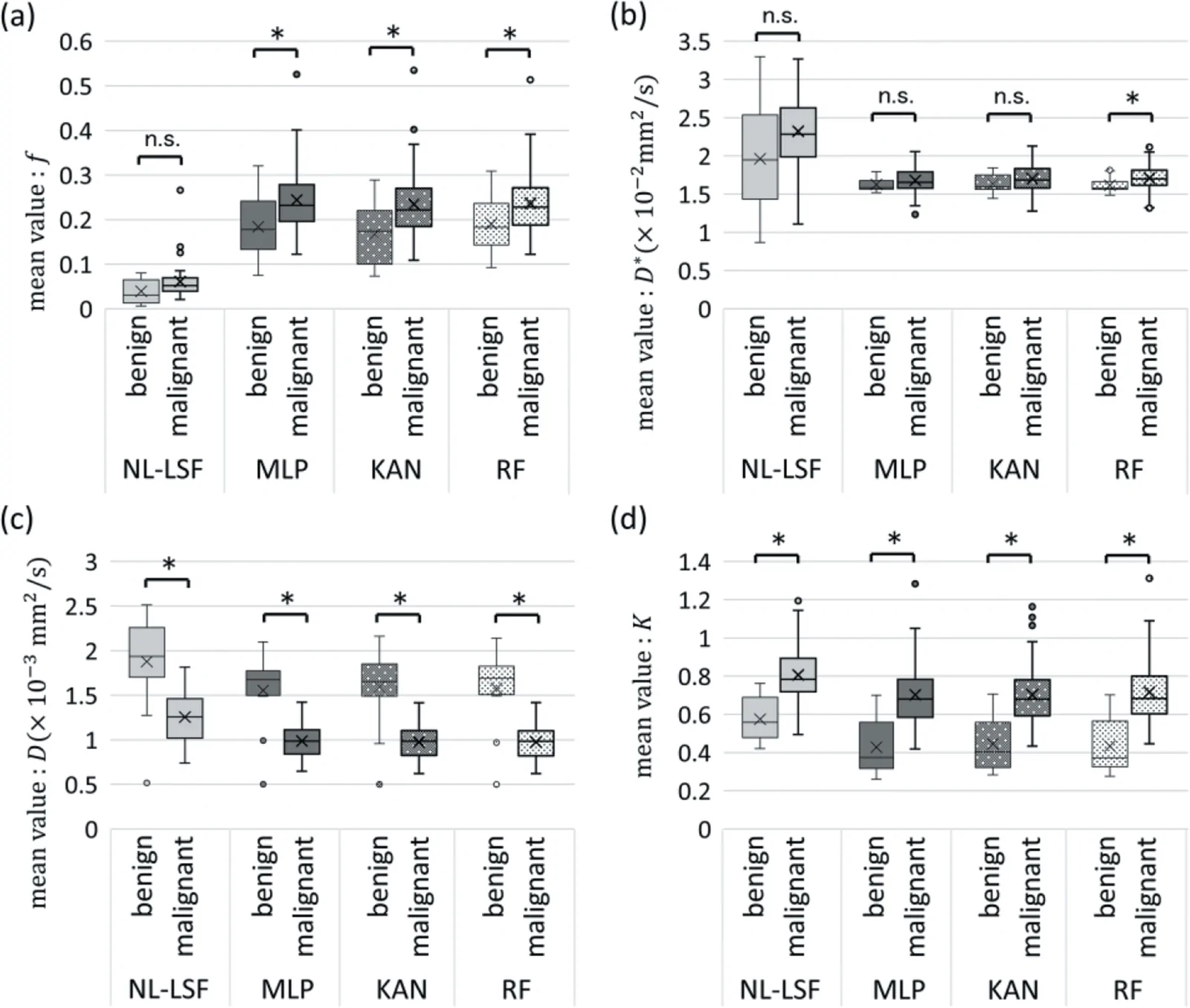
Comparison of mean values of IVIM–DKI parameters between benign and malignant lesions for NL-LSF and synQSL (MLP, KAN, and RF). Boxplots show the results for (**a**) f, (**b**) $D^{*}$, (**c**) D, and (**d**) K. *, P<0.05. IVIM–DKI, intravoxel incoherent motion–diffusional kurtosis imaging; KAN, Kolmogorov–Arnold networks; MLP, multi-layer perceptron; n.s., not significant; NL-LSF, nonlinear least squares fitting; RF, random forest; synQSL, synthetic Q-space learning.

**Fig. 7:**
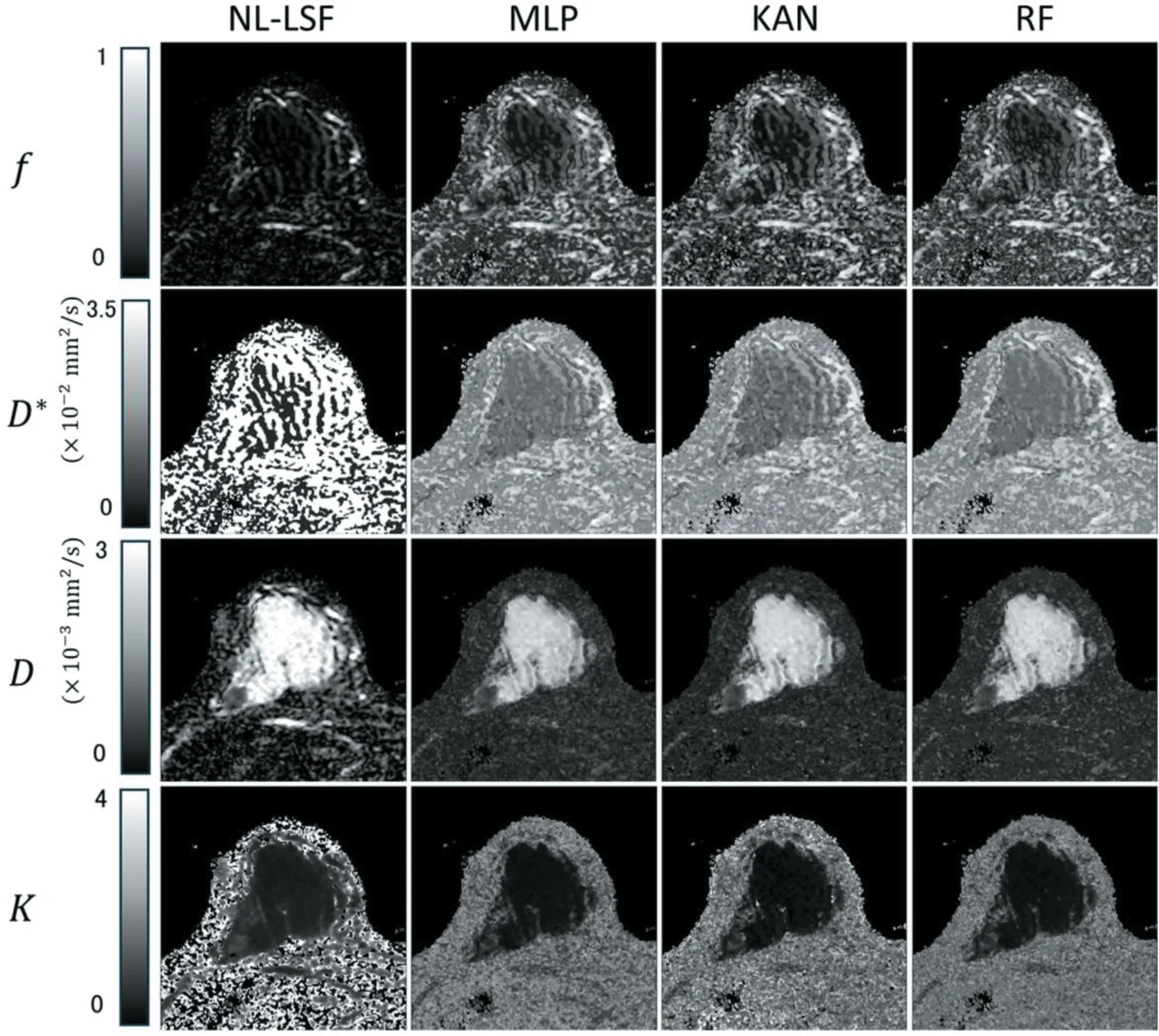
IVIM–DKI parameter maps of a representative clinical case with IDC, estimated by NL-LSF and synQSL (MLP, KAN, and RF). IDC, invasive ductal carcinoma; IVIM–DKI, intravoxel incoherent motion–diffusional kurtosis imaging; KAN, Kolmogorov–Arnold networks; MLP, multi-layer perceptron; NL-LSF, nonlinear least squares fitting; RF, random forest; synQSL, synthetic Q-space learning.

**Fig. 8:**
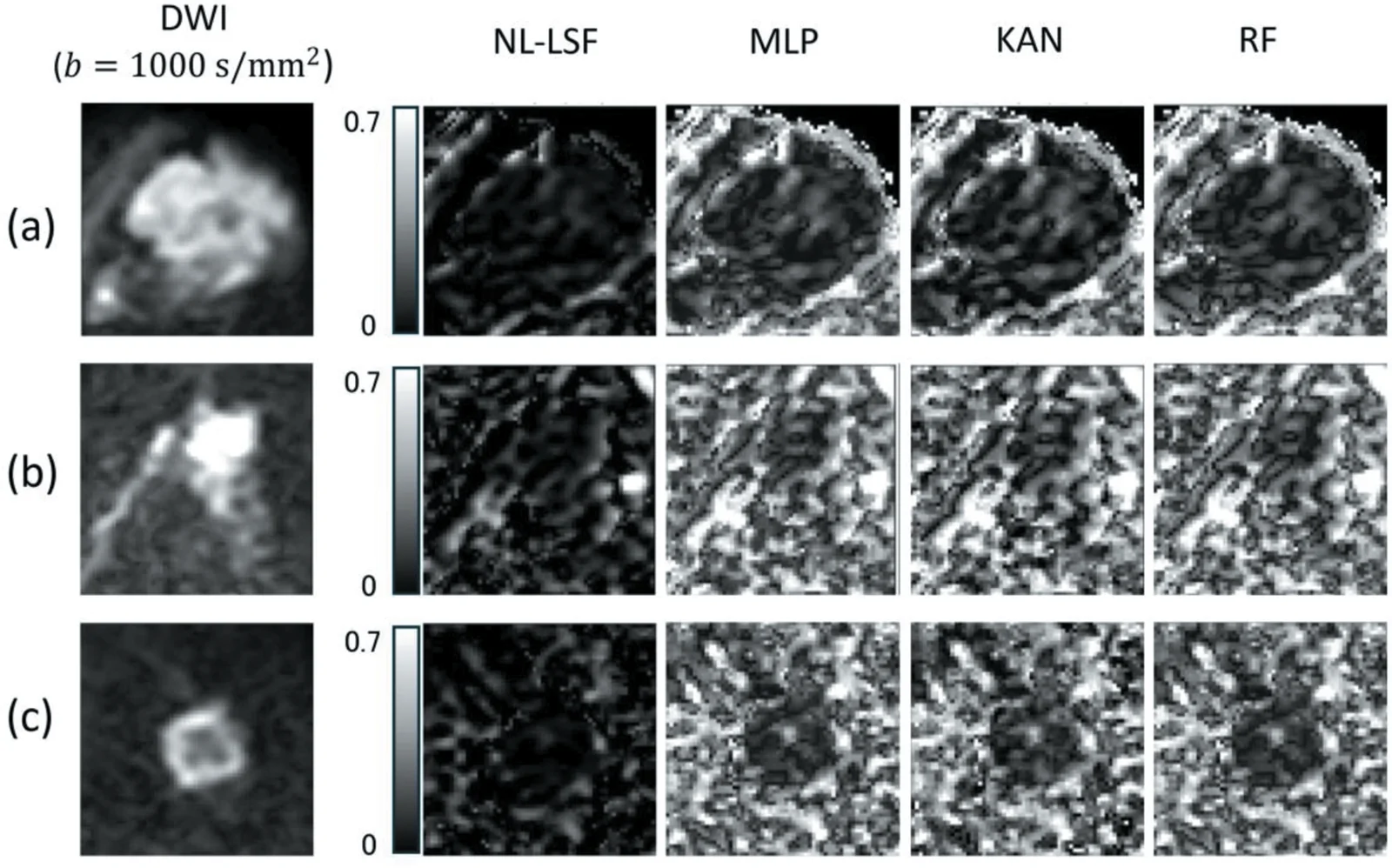
Magnified views of representative DWI ($b=1000\;s/mm^{2}$) and corresponding f map in 3 breast lesions. (**a**) Benign lesion, (**b**) DCIS, and (**c**) IDC. The parameter maps were estimated by NL-LSF and synQSL (MLP, KAN, and RF). DCIS, Ductal carcinoma *in situ*; DWI, diffusion-weighted imaging; IDC, invasive ductal carcinoma; KAN, Kolmogorov–Arnold networks; MLP, multi-layer perceptron; NL-LSF, nonlinear least squares fitting; RF, random forest; synQSL, synthetic Q-space learning.

**Table 1:** Hyperparameter search ranges

Regressor	Hyperparameter	Search range
MLP	Hidden layers	2, 3, 4, 5
	Hidden units	16, 32, 64, 128, 256
	Dropout ratio	0, 0.01, 0.1
	Batch size	1000, 10000, 100000
	Epochs	50, 100, 150, 200

KAN	Hidden layers	2, 3
	Hidden units	6, 16, 32
	grid ^1^	1, 3
	k ^2^	1, 3

RF	Number of estimators	30, 50, 100, 200, 300
	Max depth of estimators	5, 10, 20, 30

1 grid: the number of grid intervals in the domain of each activation function.

2 k: the degree of the B-spline basis functions. KAN, Kolmogorov–Arnold networks; MLP, multi-layer perceptron; RF, random forest.

## Supplementary Materials

Supplementary figures are available online.
